# Transcriptional Regulation, Epigenetic Memory, and CRISPR-Based Engineering of Combined Abiotic Stress Tolerance in Cereal Crops

**DOI:** 10.3390/biology15151249

**Published:** 2026-07-29

**Authors:** Baber Ali, Aqsa Hafeez, Nijat Imin

**Affiliations:** 1 Faculty of Engineering, Computing and Science, Western Sydney University, Penrith, NSW 2751, Australia; 2 Division of Plant Sciences, Research School of Biology, Australian National University, Acton, ACT 2601, Australia; 3 Hawkesbury Institute for the Environment, Western Sydney University, Penrith, NSW 2751, Australia

**Keywords:** combined abiotic stress, cereal crops, transcription factor networks, epigenetic stress memory, CRISPR genome editing, climate resilience

## Abstract

Cereal crops including wheat, rice, maize, barley, and sorghum collectively supply most global caloric and protein requirements, yet their productivity is increasingly constrained by combined abiotic stresses that co-occur under field conditions. Simultaneous drought, heat, salinity, and cold impose yield losses that consistently exceed those caused by individual stresses and elicit molecular responses that are qualitatively distinct from single-stress reactions and cannot be inferred from them. Despite this agronomic reality, the molecular mechanisms governing combined stress responses in cereals remain poorly resolved, and no integrated framework connecting the transcriptional, epigenetic, and genome-editing dimensions of combined stress tolerance has previously been articulated for this crop group. This review proposes a three-tier integrated framework for understanding and engineering combined abiotic stress tolerance in major cereals. The first tier encompasses transcription factor networks, including bZIP, WRKY, NAC, AP2/ERF, DREB, MYB, and HSF families, that translate combined stress signals into transcriptional reprogramming through ABA-dependent and ABA-independent pathways, hormonal crosstalk, and osmoprotectant and antioxidant defence systems. The second tier addresses the epigenetic regulatory layer, encompassing DNA methylation, histone modifications, and non-coding RNA pathways that gate TF binding site accessibility and encode stress memory in cereals. The third tier examines CRISPR-based tools, including multiplexed Cas9 editing and dCas9-based epigenome editing, that engineer validated targets from both tiers, while confronting polyploid off-target effects, growth penalties, and a laboratory-to-field validation gap. The three tiers are mechanistically coupled, with TF activity shaping epigenetic landscapes, epigenetic states gating TF access, and both providing precision engineering targets. Critical gaps include the absence of combined-stress epigenomic datasets, limited characterisation in barley and sorghum, and early-stage combined-stress-specific strategies.

## Simple Summary

Wheat, rice, maize, barley, and sorghum feed most of the world, but their harvests are increasingly damaged by weather extremes that arrive together, such as drought combined with heat, or salty soil combined with cold. When two stresses hit at once, crop damage is worse than either stress alone would cause, and plants respond in ways that differ from how they respond to a single stress. Most studies still test crops against only one stress at a time, leaving a poor understanding of how plants cope with combinations. This review brings together three areas of plant science that are usually studied separately. These are the internal switches, called regulatory proteins, that turn stress defence genes on and off, the chemical markers on plant genetic material that can remember past stress and help the plant respond faster next time, and modern gene editing tools that can fine tune these systems in crop plants. By connecting these three areas into one framework, the review shows which mechanisms are well understood, which remain unknown, and which crops need further study, particularly barley and sorghum. This work offers a roadmap for researchers and plant breeders aiming to develop cereal varieties that can withstand multiple weather stresses at once, supporting global food security as climate conditions become more unpredictable and severe.

## 1. Introduction

### 1.1. Cereals as the Foundation of Global Food Security

Cereal crops, including *Triticum aestivum* (wheat), *Oryza sativa* (rice), *Zea mays* (maize), *Hordeum vulgare* (barley), and *Sorghum bicolor* (sorghum), collectively supply approximately 40% of the total caloric and protein requirements of the human diet worldwide [1]. Rice, maize, and wheat together account for more than 60% of global food calories and underpin the nutritional security of billions of people across diverse agroecological zones [2]. Sustaining the productivity of these five cereals is therefore central to global food security, particularly because the world population is projected to exceed 9.7 billion by 2050 and total food demand is expected to increase by 35 to 56% over the same period [3]. Meeting this demand under deteriorating environmental conditions represents one of the defining agricultural challenges of the coming decades [3]. These five species were selected for this review because they collectively account for the majority of cereal-derived calories and protein consumed globally, possess the most extensively developed genomic, transcriptomic, and transformation resources among grain crops, and span an informative range of inherent stress sensitivity, from the comparatively stress-sensitive rice and wheat to the notably drought-tolerant sorghum [4,5]. Other food crops, including vegetables, fruits, and legumes, face comparable abiotic stress pressures, but their molecular stress biology and genome-editing toolkits are generally less developed than those of the major cereals, and extending the three-tier framework proposed here to these crops is an important direction for future work rather than a claim made in this review.

Global cereal production faces mounting pressure from anthropogenic climate change. The past decade has recorded the fastest rise in global mean temperature in the instrumental record, with 2023 confirmed as the warmest year in 174 years of observation [2]. Under current trajectories, global mean temperature is projected to rise by 1.2 to 1.9 °C above ambient levels during the period 2021 to 2040, and this warming will amplify the frequency, intensity, and spatial extent of abiotic stress events that directly suppress cereal yield [1]. Each degree Celsius rise in global mean temperature is estimated to reduce yield by 6.0% in wheat, 3.2% in rice, and 7.4% in maize under conditions without effective genetic adaptation [1]. Abiotic stresses including drought, heat, salinity, cold, and waterlogging are recognised as the principal environmental factors limiting cereal productivity globally, and their combined occurrence under climate change is increasingly recognised as more agronomically consequential than any single stress considered in isolation [6].

### 1.2. The Problem of Combined Abiotic Stresses in Field Conditions

Under field conditions, cereal crops rarely encounter abiotic stresses in isolation. Drought and heat co-occur with high frequency during critical reproductive stages, salinity and drought interact synergistically in irrigated production zones, and salinity and waterlogging combine in flood-prone and coastal cultivation areas [1]. The concurrent application of multiple stresses consistently produces more severe yield losses than individual stress effects considered separately, a pattern documented across wheat, rice, maize, and barley [7]. Combined drought and heat stress reduced grain yield in wheat by 56.47%, whereas individual drought and heat treatments caused losses of 44.66% and 53.05% respectively under controlled field-simulated conditions [8]. In rice, combined salinity and drought resulted in grain yield losses ranging from 53.1% to 113.3%, substantially exceeding losses attributable to either stress alone [9].

An under-recognised feature of combined stress responses is that the molecular reactions they elicit cannot be predicted from responses to individual stresses studied in isolation [10]. Transcriptomic analysis of barley under combined heat and drought stress revealed a unique and massive reprogramming of gene expression that differed qualitatively from transcriptomes generated by either stress applied alone [11]. This unpredictability reflects the activation of novel regulatory circuits, the suppression of stress-specific responses that would otherwise be adaptive, and the emergence of combinatorial signalling interactions that have no equivalent under single-stress conditions [10]. The practical consequence is that molecular targets and breeding strategies validated exclusively under single-stress conditions may not confer the expected phenotypic improvements when crops face simultaneous stresses in the field [12].

### 1.3. Limitations of Current Molecular Research Frameworks

Despite the agronomic reality of combined stress exposure, many molecular studies characterising stress tolerance mechanisms in cereals have been conducted under single-stress conditions in controlled growth environments [12], and this bias has left the regulatory logic of combined-stress responses poorly resolved [7]. Transcription factor families including bZIP, WRKY, NAC, AP2/ERF, MYB, DREB, and HSF have been characterised for their roles in responses to individual stresses such as drought, salinity, heat, and cold in rice and wheat, yet their network-level behaviour under simultaneous stress combinations remains incompletely defined [12]. Multi-omics approaches have begun to reveal the molecular regulatory networks underpinning drought responses in crops, but their application to combined stress scenarios in cereals remains limited, and the translation of omics findings into breeding applications is still at an early stage [13]. Epigenetic mechanisms have been shown to modulate stress-responsive gene expression and encode stress memory in cereals, but whether these mechanisms are specifically reprogrammed under combined stress conditions in field-relevant systems has rarely been tested directly [14]. Osmoprotectant accumulation and antioxidant enzyme activity, including proline and soluble sugar biosynthesis and the induction of superoxide dismutase, peroxidase, and catalase, are well-documented components of the single-stress response in cereals and are addressed in a dedicated subsection later in this review [15]. CRISPR-based genome editing has produced validated stress tolerance improvements in cereals through targeted editing of genes including *OsDST*, *OsERA1*, *TaDREB2*, *TaERF3*, and *ZmARGOS8*, yet most of these interventions have addressed single stress traits, and strategies for engineering tolerance to combined stresses through multiplexed or epigenome-directed editing remain at an early stage [16]. The absence of an integrated framework connecting transcriptional, epigenetic, and genome-editing dimensions of combined stress tolerance represents a significant gap between current molecular knowledge and the crop improvement outcomes the field requires [7].

This review addresses the gap identified above by proposing and developing a three-tier integrated framework for understanding and engineering combined abiotic stress tolerance in major cereal crops. The first tier encompasses the transcription factor networks that translate combined stress signals into coordinated transcriptional reprogramming, with particular focus on how TF family members interact and cooperate under simultaneous drought, heat, salinity, and cold conditions across wheat, rice, maize, barley, and sorghum. The second tier addresses the epigenetic regulatory layer that modulates TF accessibility, encodes stress memory, and may enable faster and more robust responses to recurring stress combinations [14]. The third tier examines how CRISPR-based tools, including base editing, prime editing, and emerging epigenome editing approaches, can be deployed to engineer validated targets within the first two tiers into improved cereal germplasm [17].

The conceptual advance this review provides beyond existing literature is the explicit integration of these three regulatory layers into a unified mechanistic framework applied specifically to combined stress scenarios in cereals. Existing reviews have addressed TF-mediated stress responses [12], epigenetic regulation of stress adaptation [14], and CRISPR engineering for stress tolerance [17] as separate bodies of knowledge. To the best of our knowledge, this is the first review to explicitly integrate transcription factor network dynamics, epigenetic priming, and CRISPR-based genome and epigenome editing into a single mechanistic framework for combined abiotic stress tolerance in cereals, rather than treating these three regulatory layers separately. This synthesis draws on primary experimental evidence from wheat, rice, maize, barley, and sorghum, and explicitly distinguishes between mechanisms validated in these crops and findings extrapolated from model organisms. Where direct cereal evidence is absent, this is stated and appropriate hedging applied throughout.

## 2. Combined Abiotic Stress Perception and Signalling Crosstalk in Cereals

### 2.1. Stress Perception at the Plasma Membrane

The first molecular events in the cereal stress response occur at the plasma membrane, where physical and chemical signals arising from altered osmotic pressure, elevated temperature, and ionic imbalance are detected by membrane-associated sensor proteins [18]. Osmotic stress, which is common to drought, salinity, and their combination, is sensed in part by hyperosmolality-gated calcium-permeable channels of the OSCA family [19]. In rice, *OsOSCA1.2* has been shown to detect hyperosmotic signals by responding to turgor-driven tension on the lipid bilayer, opening a calcium transport pathway that rapidly elevates cytosolic Ca^2+^ concentrations within seconds of stress onset [19]. Orthologous *OSCA* genes have been identified in wheat and maize, though their specific roles in combined osmotic and ionic stress sensing in these species remain to be established through direct functional validation [18]. Receptor-like kinases, including leucine-rich repeat receptor-like kinases, function at the plasma membrane surface to transduce extracellular stress-associated signals into intracellular phosphorylation cascades, though the specific receptor-ligand pairs responsible for heat and combined drought-heat perception in cereals remain uncharacterised [19].

Heat stress perception involves the direct effects of elevated temperature on membrane fluidity and the conformational changes in membrane-associated proteins that activate downstream signalling [20]. Under combined drought and heat conditions, both osmotic and thermal signals may be perceived simultaneously, generating overlapping and potentially competing activation signals at the plasma membrane [20]. Whether cereal cells integrate these signals at the receptor level or only at downstream convergence points remains an open question, as direct evidence for combined-stress receptor co-activation in wheat, rice, maize, barley, or sorghum is currently limited [18].

### 2.2. Reactive Oxygen Species and Calcium as Dual Second Messengers

Following plasma membrane perception, two second messenger systems, reactive oxygen species and cytosolic Ca^2+^, are rapidly activated and function in parallel and in mutual reinforcement to amplify and transduce combined stress signals [20]. ROS, primarily hydrogen peroxide and superoxide, are generated at the plasma membrane by NADPH oxidase complexes encoded by the *Rboh* gene family [21]. In rice, overexpression of *OsRbohH* enhanced tolerance to both heat and drought stress by maintaining ROS homeostasis and sustaining ABA-mediated signalling, demonstrating that OsRbohH acts as a positive regulator under combined stress conditions rather than solely as a marker of oxidative damage [21]. At low concentrations, hydrogen peroxide functions as a signalling molecule that activates downstream kinase cascades and promotes stomatal closure, while at concentrations exceeding the capacity of antioxidant scavenging systems, it triggers oxidative damage to lipids, proteins, and nucleic acids [18].

Cytosolic Ca^2+^ concentrations rise rapidly following OSCA channel activation and ROS-dependent secondary calcium release, and the resulting Ca^2+^ signal is decoded by calcium-dependent protein kinases and calcineurin B-like interacting protein kinases [18]. In rice, *OsCDPK7* enhances tolerance to combined drought and salinity by activating the expression of stress-responsive genes downstream of Ca^2+^ sensing, and *OsCPK9* improves stomatal closure and osmotic regulation under drought through a Ca^2+^-dependent mechanism [19]. In maize, *ZmCPK2* interacts with the calcium-dependent ZmCPK17 to promote stress adaptation, suggesting that CDPK-mediated Ca^2+^ decoding may involve protein–protein interactions that modulate signal output under combined stress conditions [19]. The crosstalk between ROS and Ca^2+^ signalling is bidirectional, with hydrogen peroxide promoting Ca^2+^ influx through membrane channels and Ca^2+^-activated CDPKs in turn phosphorylating and activating Rboh subunits to sustain ROS production, forming a self-amplifying loop that may intensify the combined stress signal [20]. The precise regulatory logic governing the termination of this loop under combined stress conditions in field-grown cereals remains to be established [18].

### 2.3. MAPK Cascades and ABA-Mediated Signal Transduction

Mitogen-activated protein kinase cascades constitute a major downstream transduction module activated by both ROS and Ca^2+^ signals under abiotic stress in cereals [18]. The MAPK cascade operates through sequential phosphorylation of MAPKKK, MAPKK, and MAPK components, with activated MAPKs phosphorylating transcription factors and other regulatory proteins to alter gene expression programmes [20]. In rice, *OsMPK5* is activated by multiple abiotic stresses including drought, salinity, and heat, suggesting that this kinase may function as a point of signal convergence under combined stress conditions, though its specific role under simultaneous multi-stress combinations in field conditions has not been directly tested [18]. Under combined drought and heat stress in wheat, *TaSnRK2.1* and *TaDREB2* are upregulated in an ABA-dependent manner, indicating that SnRK2-mediated phosphorylation of downstream targets including bZIP transcription factors is a component of the combined stress response in this species [22].

The canonical ABA signalling module operates through the PYR/PYL receptor proteins, which bind ABA and interact with PP2C phosphatases to release SnRK2 kinases from inhibition [19]. Activated SnRK2s phosphorylate AREB/ABF transcription factors at conserved serine residues within RXXS motifs, enabling their binding to ABRE cis-elements in the promoters of stress-responsive genes [23]. ABA concentrations are consistently elevated under combined drought, heat, and salinity stress conditions relative to single-stress treatments, and the enhanced ABA signal drives proportionally stronger SnRK2 activation and downstream transcriptional reprogramming [22]. The wheat ABA receptor gene *TaPYL4* has been shown to positively regulate drought adaptation by modulating osmotic stress-associated processes including stomatal closure and proline accumulation, though its behaviour specifically under combined stress conditions involving simultaneous heat or salinity has not been reported [19].

### 2.4. Hormonal Crosstalk Under Combined Stress Conditions

ABA does not act in isolation under combined stress conditions but engages in extensive crosstalk with jasmonic acid, salicylic acid, ethylene, gibberellic acid, brassinosteroids, and peptide hormones including C-terminally encoded peptides, whose receptor CEPR1 is conserved in barley, maize, and rice and has been linked primarily to nitrogen-demand signalling and root system architecture rather than to combined abiotic stress responses directly, with wheat *TaCEP15* shown to modulate root length and drought tolerance through a route mechanistically distinct from the canonical CEP-CEPR1 nitrogen relay [24]. The outcome of these interactions is highly context-dependent and stress-combination-specific [25]. Under combined drought and heat stress in wheat, ABA signalling is partially antagonised by jasmonic acid and ethylene signals triggered by the heat component, creating a competitive dynamic between ABA-driven stomatal closure and ethylene-mediated suppression of this response [20]. This antagonism may explain why stomatal regulation under combined drought and heat stress is less effective than under drought alone, contributing to the more severe water loss observed under the combined condition [10]. Salicylic acid can fine-tune ROS homeostasis under cold stress and may act either synergistically or antagonistically with ABA depending on the nature and intensity of the combined stress, though direct evidence for this interaction specifically in cereal crops requires further investigation [25].

Gibberellic acid is downregulated under combined drought and salinity stress, a response that reduces vegetative growth but may conserve resources for reproductive development [22]. In maize, exogenous gibberellic acid application under combined salinity and low-light stress enhanced the expression of antioxidant genes including *APX* and *GR*, reducing oxidative damage by approximately 30%, suggesting that gibberellic acid modulation may represent an adaptive strategy under combined abiotic conditions [22]. Brassinosteroids interact with ABA under combined stress in rice by influencing both stress tolerance and growth regulation through shared downstream components, though the molecular identity of the convergence points between brassinosteroid and ABA signalling specifically under combined stress conditions in cereals remains incompletely defined [25]. The complexity of hormonal crosstalk under combined stresses means that the net transcriptional output reflects a weighted integration of multiple competing and cooperating hormonal inputs rather than the activation of a single dominant pathway [20].

### 2.5. Pathway Convergence and Divergence Under Combined Stress

A defining feature of combined stress signalling in cereals is the simultaneous activation of multiple pathways that share components at certain nodes while diverging at others, producing a transcriptional output that is qualitatively distinct from that generated by any single stress [11]. The ABA-dependent pathway operates primarily through SnRK2-AREB/ABF modules, while the ABA-independent pathway activates DREB2-type transcription factors through dehydration-responsive elements without requiring elevated ABA concentrations [10]. Under combined drought and heat stress, both pathways are activated simultaneously, and DREB2-type factors, which respond to osmotic signals through the ABA-independent route, may compete with HSF-type factors activated by the heat component for binding to shared co-activators or chromatin remodelling complexes [20]. Evidence from barley transcriptomes indicates that combined heat and drought stress activates more than 900 differentially expressed transcription factor genes, far exceeding the number activated by either stress alone, consistent with the simultaneous engagement of multiple partially overlapping regulatory circuits [11].

Cold and heat represent an opposing stress combination that is increasingly relevant under climate variability, where temperature fluctuations can expose cereals to both extremes within short developmental windows [1]. The CBF/DREB1 pathway, activated under cold stress through ICE1-mediated transcriptional cascades, generally suppresses thermotolerance mechanisms, while heat activates HSF-mediated pathways that partially overlap with drought-response networks [20]. The interaction between cold-inducible CBF factors and heat-responsive pathways under simultaneously alternating temperature stress generates novel regulatory outcomes that have not been characterised in detail in any major cereal species, representing a significant knowledge gap [18].

### 2.6. Osmoprotectant Accumulation and Antioxidant Defence Systems

Alongside the signalling and transcriptional responses described above, cereals accumulate low-molecular-weight osmoprotectants and induce enzymatic antioxidants as a further layer of protection against the oxidative and osmotic components of abiotic stress [15]. Proline is the most extensively characterised osmoprotectant in cereals, and its biosynthesis is catalysed by delta-1-pyrroline-5-carboxylate synthase, encoded by *P5CS*, with degradation controlled by proline dehydrogenase. In wheat, drought stress upregulates *TaP5CS* and *TaP5CR* alongside *TaMAPK3* and *TaMAPK6*, and proline accumulation correlates with drought tolerance across contrasting genotypes, linking the MAPK cascade described in Section 2.3 to osmoprotectant biosynthesis in this species, though this evidence derives from single drought treatments rather than combined stress [26]. Soluble sugars accumulate alongside proline and contribute to osmotic adjustment and energy provision under water deficit, and both osmolyte classes are consistently reported across wheat, rice, and maize under drought and salinity, though their combined-stress dynamics in barley and sorghum remain largely uncharacterised [15].

**Figure 1:**
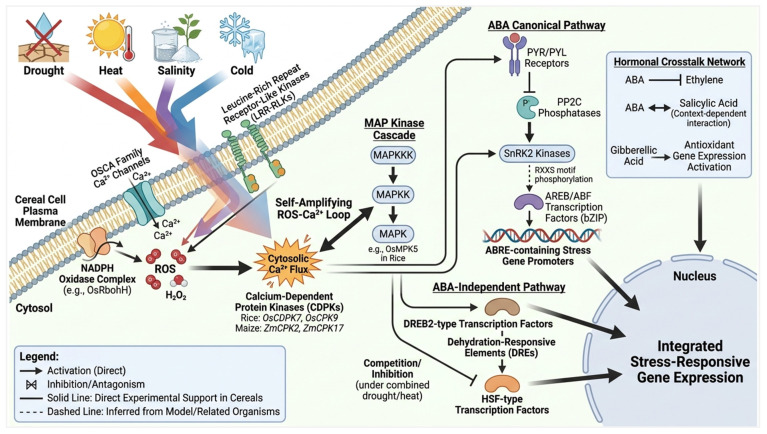
Integrated signalling framework under combined abiotic stress conditions in cereal crops. Combined abiotic stresses including simultaneous drought, heat, salinity, and cold are perceived at the plasma membrane of cereal cells through hyperosmolality-gated OSCA family calcium channels and leucine-rich repeat receptor-like kinases, which generate overlapping activation signals upon co-occurrence of multiple stress inputs [18]. Reactive oxygen species, primarily hydrogen peroxide and superoxide, are produced by NADPH oxidase complexes encoded by the *Rboh* gene family, with OsRbohH demonstrated to positively regulate ROS homeostasis under combined drought and heat stress in rice [21]. Cytosolic Ca^2+^ signals are decoded by calcium-dependent protein kinases including OsCDPK7 and OsCPK9 in rice and ZmCPK2 and ZmCPK17 in maize, generating a self-amplifying ROS-Ca^2+^ loop that intensifies the combined stress signal [19]. Mitogen-activated protein kinase cascades, including OsMPK5 in rice, transduce these second messenger signals through sequential MAPKKK-MAPKK-MAPK phosphorylation to activate downstream transcription factors [18]. The canonical ABA signalling module operates through PYR/PYL receptors, PP2C phosphatases, and SnRK2 kinases that phosphorylate AREB/ABF bZIP transcription factors at RXXS motifs to activate ABRE-containing stress-responsive gene promoters [23]. Hormonal crosstalk under combined stress involves antagonistic interactions between ABA and ethylene, context-dependent interactions between ABA and salicylic acid, and gibberellic acid modulation of antioxidant gene expression, producing a net transcriptional output that reflects a weighted integration of multiple hormonal inputs rather than activation of a single pathway [25]. The ABA-independent pathway activates DREB2-type transcription factors through dehydration-responsive elements and may compete with HSF-type factors under combined drought and heat conditions [10]. Arrows indicate activation and blunt-ended lines indicate inhibition. Pathway components supported by direct experimental evidence in the named cereal species are shown in solid lines. Components inferred from related species or model organisms are shown in dashed lines.

Enzymatic antioxidants, principally superoxide dismutase, peroxidase, catalase, and ascorbate peroxidase, detoxify the reactive oxygen species described in Section 2.2 and limit lipid, protein, and nucleic acid damage under stress [15]. In rice, superoxide dismutase and ascorbate peroxidase activities were more strongly induced under combined drought and heat stress than under either stress alone, while catalase induction was comparatively stress-specific, indicating that the antioxidant response to combined stress in this species is not a simple summation of single-stress responses and instead involves enzyme-specific regulatory logic that parallels the non-additive transcriptional patterns described in Section 2.5 [27]. In maize, CRISPR/Cas9 knockout of *ZmPP2C15* reduced superoxide dismutase, peroxidase, ascorbate peroxidase, and catalase activities together with proline content under drought stress, while overexpression in the heterologous *Arabidopsis* system enhanced these same traits, indicating that ZmPP2C15 positively couples ABA-related phosphatase signalling to antioxidant enzyme induction in maize under single-stress drought conditions, with its behaviour under combined stress not yet reported [28]. These osmoprotectant and antioxidant systems are downstream transcriptional targets of several TF families discussed in Section 3, including DREB, NAC, and MYB members, and the regulatory connections between TF activation and osmoprotectant or antioxidant gene induction under combined stress in cereals remain an open area for functional validation rather than a mechanism yet demonstrated in these species. The signalling architecture described across Section 2.1, Section 2.2, Section 2.3 and Section 2.4 provides the mechanistic foundation for the transcription factor networks discussed in Section 3, where the identity, interactions, and combined-stress behaviour of specific TF families are examined in detail. The convergence of ROS, Ca^2+^, MAPK, and hormonal cascades under representative combined stress combinations in cereals is illustrated in Figure 1.

## 3. Transcription Factor Networks Mediating Combined Stress Responses in Cereals

### 3.1. Overview of Transcription Factor Families in Combined Stress Responses

Transcription factors regulate the expression of stress-responsive genes by binding to specific cis-regulatory elements in gene promoters and activating or repressing transcription in response to upstream signalling events [12]. In cereals, the signalling cascades described in Section 2 converge on a set of TF families that collectively determine the transcriptional output under combined stress conditions. The major families implicated in abiotic stress responses in cereals include bZIP, WRKY, NAC, AP2/ERF, DREB, MYB, and HSF, each characterised by a distinct DNA-binding domain, a specific set of cis-regulatory targets, and partially overlapping but non-identical stress specificities [12]. Under combined stress conditions, multiple TF families are activated simultaneously, and their interactions, competitive binding dynamics, and cooperative regulatory relationships determine the nature and amplitude of the transcriptional reprogramming [29]. Table 1 provides a summary of the key TF families, their validated gene members in major cereals, the stress combinations they address, and the experimental systems in which their functions have been confirmed.

### 3.2. bZIP and AREB/ABF Transcription Factors

The bZIP family represents the primary ABA-dependent transcriptional output module in cereals, operating downstream of the SnRK2-mediated phosphorylation events described in Section 2.3 [23]. AREB and ABF subfamily members of bZIP proteins bind to ABA-responsive elements containing the ACGT core motif in the promoters of stress-responsive genes, activating transcription of genes encoding late embryogenesis abundant proteins, dehydrins, osmoprotectant biosynthesis enzymes, and antioxidant system components [32]. In rice, *OsbZIP23* confers ABA-dependent drought and salinity tolerance when overexpressed, and its activity depends on SnRK2-mediated phosphorylation at multiple serine residues within conserved RXXS motifs [32]. *OsbZIP71* confers both salinity and drought tolerance in rice by directly binding to the promoters of stress-responsive target genes, demonstrating that a single bZIP member can integrate responses to distinct ionic and osmotic stress signals [32]. In wheat, *TaAREB3* overexpression enhances ABA sensitivity and drought resistance, and *TaFDL2-1A* and *TabZIP8-7A* form a transcriptional activation complex that synergistically promotes the expression of ABA-induced genes under drought stress [32]. The formation of homo- and heterodimers between bZIP proteins through their leucine zipper domains is a critical determinant of DNA-binding specificity and transcriptional output, and the combinatorial diversity of bZIP dimerisation may allow fine-tuned responses to different stress combinations [32]. In maize, *ZmbZIP68* inhibits *DREB1* expression and acts as a negative regulator in the CBF-dependent cold signalling pathway, revealing that bZIP members can modulate the ABA-independent cold response and that cross-regulatory interactions between bZIP and DREB pathways occur at the transcriptional level [32]. The bZIP dimerisation patterns that predominate under combined drought-heat or salinity-cold stress in cereals have not been systematically characterised, and direct in planta investigation is needed [12].

### 3.3. WRKY Transcription Factors

WRKY TFs are defined by a conserved WRKYGQK heptapeptide sequence in their DNA-binding domain and bind predominantly to W-box cis-elements with the core sequence TTGACC/T in the promoters of target genes [29]. The WRKY family is one of the largest TF families in cereals, with over 100 members identified in rice and wheat, and multiple members are induced by drought, salinity, heat, and their combinations [29]. In maize, *ZmWRKY40* is rapidly and strongly induced by drought, high salinity, high temperature, and ABA, with expression levels increasing more than tenfold within one hour of drought treatment, suggesting that this factor may function as an early integrator of multiple stress signals [29]. Overexpression of *ZmWRKY82* in transgenic maize conferred enhanced drought stress tolerance by modulating ROS homeostasis and ABA signalling, with transgenic lines exhibiting improved survival rates and reduced oxidative damage compared with wild-type plants under water deficit conditions [34]. In rice, inactivation of *OsWRKY5* through a loss-of-function mutation enhanced drought tolerance by modulating ABA signalling and stomatal behaviour, demonstrating that WRKY factors can act as negative regulators of stress tolerance and that their suppression may improve combined-stress performance [35]. In wheat, *TaWRKY1-2D* overexpression enhanced drought tolerance by upregulating antioxidant and stress-responsive genes including *TaP5CS*, *TaPOD*, and *TaCAT* (the osmoprotectant and antioxidant genes introduced in Section 2.6), and yeast two-hybrid analysis identified *TaDHN3* as an interaction partner, suggesting that post-translational regulation of dehydrin proteins may be part of the WRKY-mediated drought response in this species [36]. Whether WRKY TFs integrate combined drought and heat signals simultaneously, rather than responding sequentially to each component, has not been tested directly in field-grown cereals [18].

### 3.4. NAC Domain Transcription Factors

NAC TFs are defined by a conserved N-terminal NAC domain comprising approximately 150 amino acids that mediate DNA binding and dimerisation, and they regulate target genes through binding to NAC recognition sequences in gene promoters [12]. NAC factors participate in both ABA-dependent and ABA-independent stress response pathways and have been validated as positive and negative regulators of drought, salinity, and heat tolerance in rice, wheat, and maize [12]. In rice, knockout of *OsNAC092* promoted drought tolerance by enhancing stomatal regulation and reducing water loss, demonstrating that this NAC member acts as a negative regulator of the drought stress response and that its suppression is a viable strategy for improving drought performance in rice [37]. *OsNAC25* was induced by low temperature, high temperature, and salinity within 12 h of stress application in rice, and its overexpression reduced oxidative damage and improved survival under severe drought at the vegetative stage by coordinating phenylpropanoid biosynthesis and hormone signal transduction pathways [38]. In wheat, overexpression of *TaNAC071-A* enhanced water use efficiency and drought tolerance by improving stomatal regulation, while knockout of the same gene decreased drought tolerance, demonstrating that this NAC member is a positive regulator of the drought response in this hexaploid species [12]. Functional characterisation of NAC TFs under combined stress in barley and sorghum lags well behind rice and wheat, and how far NAC-mediated mechanisms are conserved across all five cereals is still an open question [12].

### 3.5. AP2/ERF and DREB Transcription Factors

The AP2/ERF superfamily is one of the largest and most functionally diverse TF families in plants, characterised by a conserved AP2 DNA-binding domain of approximately 60 amino acids [43]. Within this superfamily, DREB subfamily members bind to DRE/CRT cis-elements with the core sequence TACCGACAT and activate stress-responsive genes through an ABA-independent pathway, while ERF subfamily members preferentially bind GCC box elements and regulate ethylene-responsive and stress-associated gene expression [43]. In wheat, a genome-wide analysis identified 630 putative AP2/ERF superfamily genes, with multiple members showing strong induction under heat stress conditions and containing ABRE, DRE, and MYB cis-elements in their promoters, indicating their capacity to integrate both ABA-dependent and ABA-independent signals [43]. Under combined drought and heat stress in wheat, transcriptome profiling at three key spike developmental stages identified *TaEREBP1-L* as a combined-stress-specific ERF that operates through ABA-independent hormonal and osmotic regulation pathways distinct from the canonical ABA-dominated response, and *DREB2A* and *DREB2C* were identified as regulators of *HSFA3*-like genes, revealing cross-regulation between thermotolerance and dehydration response networks [40]. DREB2 homologs have been confirmed in wheat, rice, and maize, where they respond to both osmotic and heat signals, making them candidates for engineering tolerance to combined drought-heat stress, though constitutively active forms of these factors produce growth penalties that require careful management in breeding applications [10]. How DREB2-type and ERF-type factors interact under combined drought and salinity stress in barley and sorghum remains uncharacterised and is a priority for future functional genomics work [18].

### 3.6. MYB and HSF Transcription Factors

MYB TFs are characterised by a conserved MYB DNA-binding domain containing one to three imperfect repeats of approximately 52 amino acids each and regulate a diverse range of processes including anthocyanin biosynthesis, cell wall composition, and abiotic stress responses [12]. In maize, comparative transcriptome analyses under combined drought, salinity, heat, and cold stress identified MYB and NAC TFs among the most consistently modulated regulatory genes, with MYB family members associated with carbohydrate synthesis, cell wall remodelling, and protein ubiquitination under combined stress conditions [7]. Specific MYB genes are less well characterised under combined stress than WRKY or NAC members, particularly in wheat, barley, and sorghum [12].

HSF TFs are defined by a conserved helix-turn-helix DNA-binding domain that recognises heat shock elements with the consensus sequence nGAAn in the promoters of heat shock protein genes [12]. In maize, ectopic overexpression of *ZmHsf05* in rice conferred drought tolerance through enhanced proline accumulation, reduced leaf water loss, and modulation of ABA signalling, demonstrating that HSF function extends beyond heat stress to encompass drought responses even when expressed in a different cereal species [41]. In wheat, *TaHSFC3B* enhanced drought tolerance through ROS scavenging and ABA pathway modulation, and its expression was induced by both polyethylene glycol treatment and ABA, indicating that HSF members in wheat can integrate drought and hormonal signals independently of heat [42]. This dual heat- and drought-responsive capacity suggests HSF members may act as convergence nodes for combined heat-drought signalling, though the molecular basis of this dual specificity has not been tested directly in field-grown cereals [20].

### 3.7. TF Network Interactions and Combined Stress Regulation

**Figure 2:**
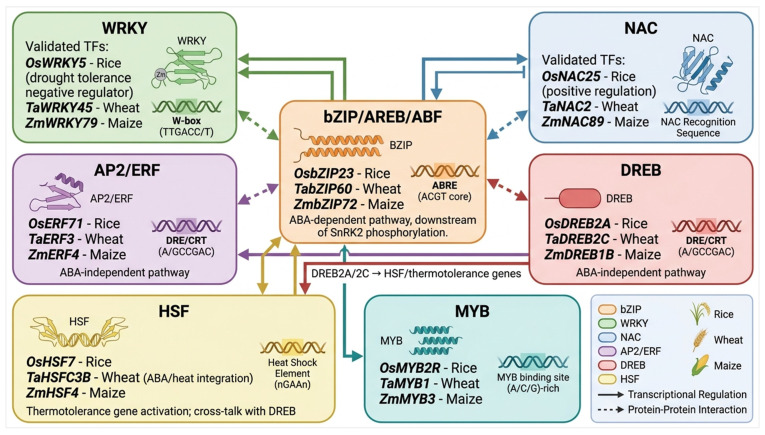
Transcription factor regulatory networks mediating combined abiotic stress responses in major cereal crops. Seven major TF families activated under combined abiotic stress conditions in cereals are depicted, showing their DNA-binding domain specificities, cis-regulatory element targets, and inter-family regulatory interactions. bZIP and AREB/ABF factors bind ABRE cis-elements containing the ACGT core motif and operate downstream of SnRK2-mediated phosphorylation in the ABA-dependent pathway, with OsbZIP23 and OsbZIP71 validated in rice and TaAREB3 and TabZIP8-7A validated in wheat [32]. WRKY factors bind W-box elements with the TTGACC/T core sequence, with ZmWRKY40 and ZmWRKY82 validated as multi-stress integrators in maize, OsWRKY5 as a negative regulator of drought tolerance in rice, and TaWRKY1-2D as a positive regulator in wheat [29,34,35,36]. NAC domain proteins bind NAC recognition sequences through their conserved N-terminal domains, with OsNAC092 acting as a negative regulator of drought tolerance in rice, OsNAC25 as a positive regulator induced by temperature, salinity, and drought in rice, and TaNAC071-A as a positive regulator of water use efficiency in wheat [37,38]. AP2/ERF and DREB factors bind DRE/CRT elements through the ABA-independent pathway, with TaEREBP1-L identified as a combined-stress-specific ERF in wheat and DREB2A and DREB2C shown to regulate HSF-mediated thermotolerance genes, revealing cross-regulation between dehydration and heat response networks [40,43]. HSF factors bind heat shock elements with the nGAAn consensus sequence, with ZmHsf05 shown to confer drought tolerance in rice and TaHSFC3B demonstrated to integrate drought and ABA signals independently of heat in wheat [41,42]. MYB factors are associated with combined stress responses in maize through modulation of carbohydrate synthesis and cell wall remodelling pathways [7]. Network edges depict experimentally validated protein–protein interactions and transcriptional regulatory relationships. Species labels indicate the cereal in which each factor has been directly validated.

A defining challenge in understanding combined stress responses in cereals is that individual TF families do not operate independently but form regulatory networks through physical interactions, competitive binding to shared cis-elements, and transcriptional cascades in which one TF regulates the expression of another [12]. Meta-transcriptomic analysis of wheat under multiple abiotic stresses identified BES1/BZR1 brassinosteroid signalling TFs as hub genes integrating responses to heat, cold, drought, and salt stress simultaneously, suggesting that brassinosteroid-responsive TFs may coordinate multi-stress responses in wheat beyond their established roles in growth regulation [44]. In barley under combined heat and drought stress, more than 900 transcription factor genes were differentially expressed, encompassing members of the WRKY, NAC, MYB, bZIP, and HSF families simultaneously, and the stress-tolerant cultivar Otis showed a substantially different TF activation pattern compared with the stress-sensitive cultivar Golden Promise, indicating that the composition and connectivity of the TF network rather than the activation of individual factors determines the tolerance phenotype [11]. The interaction between DREB2-type ABA-independent factors and HSF-type heat-responsive factors under combined drought and heat stress, where both may compete for binding to shared co-activators or differ in chromatin accessibility at target promoters, represents an important but incompletely characterised regulatory interface in cereals [40]. The transcriptional regulatory complexity described across Section 3.2, Section 3.3, Section 3.4, Section 3.5 and Section 3.6 raises a critical question about how these TF networks are modulated at the chromatin level, which is addressed in Section 4 through examination of the epigenetic mechanisms that control TF binding site accessibility and encode stress memory in cereals. Figure 2 illustrates the key TF families, their regulatory interactions, and their combined-stress target gene networks in major cereals.

## 4. Epigenetic Regulation and Stress Memory in Cereals

### 4.1. Overview of Epigenetic Regulation in Cereal Stress Responses

The TF networks described in Section 3 do not operate on a static chromatin template but function within a dynamically regulated epigenetic landscape that determines which gene promoters are accessible for TF binding and which are occluded by compact chromatin structure [45]. Epigenetic mechanisms encompass heritable changes in gene expression that do not involve alterations to the underlying DNA sequence, and they operate through three major interacting systems, namely DNA methylation, histone post-translational modifications, and non-coding RNA pathways [46]. In cereals, these three systems respond to abiotic stress by remodelling chromatin at stress-responsive loci, enabling rapid and coordinated transcriptional reprogramming that complements and amplifies TF-mediated regulation [14]. A particularly important feature of epigenetic regulation is its capacity to encode stress memory, whereby a prior stress exposure leaves persistent chromatin marks that prime faster and stronger transcriptional responses to subsequent stress encounters [47]. Whether these memory mechanisms are specifically engaged under combined stress in cereals is addressed, with the evidence gap flagged explicitly, throughout this section. Table 2 summarises the key epigenetic regulators, their target loci, the stress conditions under which they have been characterised, and the cereal species in which direct evidence exists.

### 4.2. DNA Methylation Dynamics Under Abiotic Stress

DNA methylation in cereals occurs predominantly at cytosine residues in three sequence contexts, CG, CHG, and CHH, and is established and maintained by distinct methyltransferases including MET1 for CG contexts, CMT3 for CHG contexts, and DRM2 for CHH contexts via the RNA-directed DNA methylation pathway [46]. Stress-induced changes in DNA methylation can either activate or repress stress-responsive genes depending on whether methylation occurs in promoter regions or gene bodies, and the direction of the methylation change is locus-specific and stress-type-specific [48]. In rice, whole-genome bisulphite sequencing of cultivars with contrasting drought and salinity stress responses revealed extensive differences in DNA methylation patterns, with numerous differentially methylated regions associated with differential expression of stress-response genes, and transposon-associated differentially methylated regions coupled to the transcript abundance of nearby protein-coding genes [48]. In maize, drought stress increased DNA methylation levels genome-wide, and the methylomes of drought-tolerant inbred lines were substantially more stable under water stress than those of drought-sensitive lines, suggesting that epigenome stability may be a component of drought tolerance in this species [48]. In wheat, DNA methylation in the promoter of *NAC111* resulted in increased drought sensitivity through repression of this stress-responsive gene, demonstrating that promoter methylation can act as a negative regulator of drought tolerance when it silences positive regulatory factors [22]. Drought stress induced upregulation of multiple DNA methyltransferase genes in wheat, and these changes were associated with altered expression of genes involved in ion homeostasis under combined drought and salinity conditions [22]. The RNA-directed DNA methylation pathway, which deposits CHH methylation through 24-nucleotide small interfering RNAs generated by RNA polymerase IV and processed by DICER-LIKE 3, plays an important role in silencing transposable elements near stress-responsive genes, and perturbation of this pathway under heat stress can lead to transposable element reactivation and altered expression of neighbouring genes [46]. Whether combined stress reprograms the DNA methylation landscape in a qualitatively distinct way relative to individual stresses has not been tested directly in field-grown cereals and remains a priority for epigenomic research in this field [45].

### 4.3. Histone Modifications and Chromatin Remodelling

Histone post-translational modifications regulate chromatin accessibility and transcriptional activity through a diverse array of covalent marks on the N-terminal tails of core histone proteins [46]. The activating mark H3K4me3, deposited by SET domain group methyltransferases, is associated with transcriptionally active chromatin and accumulates at the promoters and transcription start sites of stress-responsive genes during drought, salinity, and heat stress in cereals [50]. The repressive mark H3K27me3, deposited by Polycomb Repressive Complex 2, maintains gene silencing under non-stress conditions and its removal from stress-responsive gene loci is required for transcriptional activation under stress [50]. In rice, the H3K4 methyltransferase SDG721 binds to and deposits H3K4 marks in the promoter and coding region of *HKT1;5*, a sodium transporter gene, to regulate its expression under saline-alkaline stress, providing a direct mechanistic link between histone methylation and ion homeostasis in a major cereal [50]. Histone acetylation, catalysed by histone acetyltransferases and removed by histone deacetylases, promotes an open chromatin state that facilitates TF binding by neutralising the positive charge on lysine residues and reducing DNA-histone electrostatic interactions [46]. In maize, H3K9ac levels were significantly increased in the promoter regions of *Hsf-01* and *Hsf-15* under high temperature stress, providing a direct link between heat-stress-responsive histone acetylation and HSF gene activation in a cereal crop [50]. Heat stress decreases H3K9me2 levels in rice, a modification associated with heterochromatin maintenance, and this reduction is linked to the derepression of heat-responsive gene loci [45]. Bivalent chromatin domains carrying both the activating H3K4me3 mark and the repressive H3K27me3 mark at the same locus have emerged as important regulators of stress-responsive gene expression, allowing genes to be maintained in a poised state that enables rapid activation upon stress perception while remaining repressed under non-stress conditions [50]. Dynamic switching between bivalent and monovalent chromatin states under combined drought-heat or salinity-cold conditions has not been characterised in any major cereal, leaving a significant gap in how the epigenome integrates simultaneous stress signals [47].

### 4.4. Non-Coding RNA Regulation and Stress Memory

Non-coding RNAs including microRNAs, small interfering RNAs, and long non-coding RNAs constitute a third epigenetic regulatory layer that modulates stress-responsive gene expression post-transcriptionally and at the chromatin level in cereals [53]. MicroRNAs of approximately 21 nucleotides bind to complementary sequences in target mRNAs to direct their cleavage or translational repression, and multiple miRNA families are differentially regulated under drought, heat, salinity, and cold stress in rice, wheat, and maize [53]. In wheat, small RNAs and their target genes are associated with the transgenerational effects of water-deficit stress, with specific miRNA populations persisting in progeny plants and contributing to altered stress-responsive gene expression in the next generation [47]. Small interfering RNAs direct the RNA-directed DNA methylation pathway to silence transposable elements and certain protein-coding genes under stress, and in maize and rice, siRNA-directed methylation maintains euchromatin-heterochromatin boundaries near stress-responsive genes, with drought disturbing these boundaries in genotype- and tissue-dependent ways [47]. Long non-coding RNAs of more than 200 nucleotides regulate stress-responsive gene expression through multiple mechanisms, including recruitment of chromatin-modifying complexes such as Polycomb Repressive Complex 2 to target loci, acting as molecular sponges that sequester specific miRNAs, and modulating three-dimensional chromatin architecture through R-loop formation [53]. In rice, the lncRNA *TCONS_00028567* participates in short-term drought stress memory, suggesting that lncRNA-mediated chromatin regulation may be a component of the stress priming response in this cereal [47]. lncRNA roles under combined abiotic stress are far less characterised than under individual stresses, and the potential for lncRNA-mediated coordination of epigenetic responses to simultaneous drought and heat or salinity and cold in wheat, maize, barley, and sorghum remains to be established [14].

### 4.5. Somatic Stress Memory and Epigenetic Priming

Somatic stress memory refers to the ability of a plant that has experienced a prior stress to mount a faster and more robust transcriptional response upon re-exposure to the same or a related stress within the same generation, and this capacity has been linked to the persistence of specific histone modification patterns at stress-responsive gene loci after the initial stress subsides [47]. The molecular basis of somatic stress memory in plants involves the accumulation of phosphorylated RNA polymerase II at the promoters of memory genes during the initial stress, and the maintenance of histone marks including H3K4me3 and specific histone acetylation patterns that keep chromatin in a primed, accessible state during the inter-stress recovery period [47]. In cereals, somatic stress memory has been most extensively studied in the context of drought priming, where prior mild drought exposure improves the performance of rice, wheat, and maize plants under subsequent severe drought, though the specific epigenetic marks responsible for this improvement and their molecular maintenance mechanisms have not been fully characterised in all three species [54]. Barley relies on small RNA-mediated regulation to control the expression of drought-responsive genes under salinity and drought conditions, and sorghum exhibits dynamic changes in histone modifications that enhance drought tolerance through prolonged stress exposure, suggesting that stress memory mechanisms may operate across multiple cereal species [22]. Whether drought-established somatic memory improves responses to a qualitatively different subsequent stress, or to a combined-stress scenario, has not been tested directly in cereals, a question directly relevant to designing field stress-priming strategies [47].

### 4.6. Transgenerational Stress Memory

**Figure 3:**
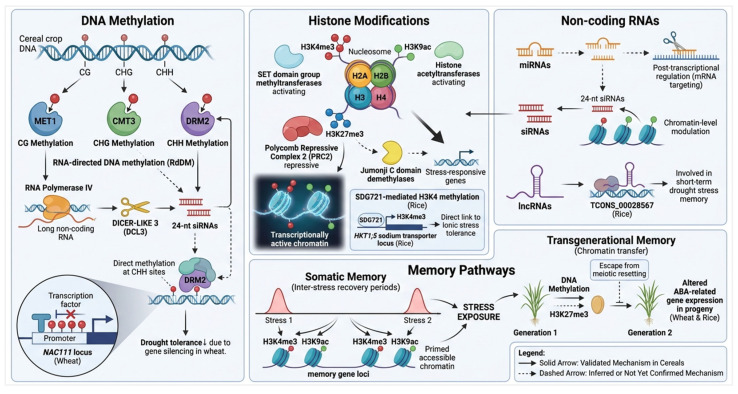
Epigenetic mechanisms underlying stress memory encoding and retrieval in cereal crops. Three major epigenetic regulatory systems that modulate stress-responsive gene expression and encode stress memory in cereals are depicted. DNA methylation at CG, CHG, and CHH contexts is established and maintained by MET1, CMT3, and DRM2 methyltransferases, respectively, with the RNA-directed DNA methylation pathway depositing CHH marks through 24-nucleotide siRNAs generated by RNA polymerase IV and processed by DICER-LIKE 3 [46]. Stress-induced changes in promoter methylation regulate TF access to stress-responsive gene loci, with promoter methylation of NAC111 demonstrated to reduce drought tolerance in wheat through gene silencing [22]. Histone modifications include the activating marks H3K4me3 deposited by SET domain group methyltransferases and H3K9ac deposited by histone acetyltransferases, both associated with transcriptionally active stress-responsive chromatin, and the repressive mark H3K27me3 deposited by Polycomb Repressive Complex 2, whose removal by Jumonji C domain demethylases is required for stress gene activation [50]. SDG721-mediated H3K4 methylation at the HKT1;5 sodium transporter locus in rice provides a direct mechanistic link between histone methylation and ionic stress tolerance [50]. Non-coding RNA pathways including miRNAs, siRNAs, and lncRNAs modulate stress-responsive gene expression post-transcriptionally and at the chromatin level, with lncRNA TCONS_00028567 shown to participate in short-term drought stress memory in rice [47]. Somatic stress memory involves persistence of H3K4me3 and histone acetylation marks at memory gene loci during inter-stress recovery periods, maintaining chromatin in a primed accessible state [47]. Transgenerational stress memory involves inheritance of DNA methylation and H3K27me3 marks that escape meiotic resetting, with stress-induced epigenetic changes shown to alter ABA-related gene expression in progeny plants in wheat and rice [54]. Solid arrows indicate direct experimentally validated mechanisms in cereals. Dashed arrows indicate mechanisms inferred from related species or requiring further cereal-specific validation.

Transgenerational stress memory involves the inheritance of epigenetic marks that escape meiotic resetting and are transmitted to progeny plants that have not themselves experienced the inducing stress [54]. The heritable modifications responsible for transgenerational memory include DNA methylation maintained by MET1 or DRM2, histone marks such as H3K27me3, and the activity of the RNA-directed DNA methylation pathway [54]. In wheat and rice, specific DNA methylation patterns and histone modifications established during drought or salt stress have been shown to persist in germline cells, altering ABA-related and auxin-related gene expression in the next generation, though the proportion of stress-induced epigenetic changes that escape meiotic resetting versus those that are reset during germline development remains to be fully quantified in these species [54]. Heat stress in wheat has been shown to induce transgenerational tolerance to subsequent high temperature stress, with molecular analysis indicating that changes in gene expression and protein accumulation in the primed generation are maintained in progeny plants, though the precise epigenetic marks responsible for this transmission have not been identified [45]. The reproductive developmental stages of cereals, encompassing pollen development, fertilisation, and early seed development, are particularly sensitive to abiotic stresses and represent critical windows during which epigenetic reprogramming can either transmit or reset stress-induced chromatin marks [55]. During male gametophyte development in cereals, DNA methylation and small RNA pathways are dynamically regulated, and stress-induced perturbations of these pathways during pollen formation may contribute to transgenerational epigenetic effects [55]. Whether transgenerational stress memory remains stable across multiple field generations, and whether it can be deliberately induced and maintained in breeding programmes to improve combined stress tolerance, has not been tested through direct multi-generational field experiments [54]. The epigenetic regulatory mechanisms described in this section modulate TF network function by determining the accessibility of TF binding sites across the cereal genome, and the mechanistic connections between these two regulatory tiers are the subject of Section 5. Figure 3 illustrates the major epigenetic mechanisms, their target loci, and their roles in stress memory encoding and retrieval in cereal crops.

## 5. Interconnections Between Transcriptional and Epigenetic Layers

### 5.1. The Mechanistic Basis of TF-Epigenetic Crosstalk

The TF networks described in Section 3 and the epigenetic regulatory mechanisms described in Section 4 do not operate as independent layers but are mechanistically coupled through bidirectional interactions that operate at the level of individual gene promoters and across the genome [56]. TFs influence the epigenetic landscape by recruiting chromatin-modifying enzymes and remodelling complexes to specific genomic loci, while epigenetic marks determine the accessibility of TF binding sites and thereby govern which TF-driven transcriptional programmes can be activated under any given stress condition [46]. This bidirectional coupling means that the transcriptional output of the stress response is not determined solely by TF activation states or solely by chromatin configuration but emerges from the integration of both layers simultaneously [45]. In cereals, TF-epigenetic crosstalk has been characterised for only a small number of specific TF-chromatin modifier pairs, all under individual stress conditions. The broader architecture of these interactions under combined stress remains largely uncharacterised [18]. Table 2 includes the key TF-chromatin modifier interactions identified in cereals to date, alongside the stress conditions and species in which direct experimental evidence exists.

### 5.2. TF Recruitment of Histone-Modifying Enzymes

One of the primary mechanisms through which TFs direct epigenetic changes at stress-responsive loci is the physical recruitment of histone acetyltransferases and histone deacetylases to specific gene promoters, thereby locally altering histone acetylation states and chromatin accessibility [56]. In rice, the drought-responsive transcription factor OsWR2 physically recruits the histone deacetylase HDA704 to the promoter of *OsABI5*, an ABA-responsive gene, where HDA704 catalyses deacetylation of histone H4 at lysine 8, reducing chromatin accessibility at this locus and modulating ABA-dependent gene expression under drought conditions [51]. This example demonstrates that TF-directed histone deacetylation can act as a regulatory mechanism for fine-tuning the amplitude of ABA-responsive transcription under drought stress, and it illustrates how a single TF-HDAC interaction can integrate stress signalling with chromatin-level gene regulation at a specific downstream target [51]. Conversely, TFs also recruit histone acetyltransferases to promote chromatin opening at stress-responsive gene promoters. In wheat, the histone acetyltransferase GCN5 interacts with the NAC-like transcription factor NACL and the heat shock transcription factor HsfA1 to regulate the expression of thermotolerance genes including *G1* and *PSBR1* under heat stress, establishing a direct link between TF-mediated stress recognition and HAT-dependent chromatin activation in a major cereal [56]. The functional conservation of GCN5-mediated thermotolerance between wheat and *Arabidopsis thaliana* has been demonstrated through complementation experiments in which *TaGCN5* restored heat tolerance to *Arabidopsis gcn5* mutant plants, though this represents evidence of functional homology between species rather than direct validation of the same mechanism operating in field-grown wheat under combined stress conditions [56]. In rice, cold stress induces hyperacetylation of H3K9 at the promoter and upstream region of *OsDREB1b*, promoting chromatin remodelling and enhanced transcriptional activation of this cold-responsive DREB factor, and this HAT-mediated chromatin opening is required for full induction of *OsDREB1b* under cold stress [56]. Whether similar TF-HAT and TF-HDAC recruitment operates under combined stress, where multiple TF families compete simultaneously for HAT and HDAC access, has not been tested through chromatin or co-immunoprecipitation analyses under combined-stress treatments [45].

### 5.3. TF-Directed Chromatin Remodelling Complex Activity

Beyond histone-modifying enzymes, TFs in cereals recruit ATP-dependent chromatin remodelling complexes that physically reposition or evict nucleosomes to alter DNA accessibility at target gene promoters [46]. The SWI/SNF chromatin remodelling complex is among the most extensively studied in plants and functions by using ATP hydrolysis to slide or eject nucleosomes, creating open chromatin regions that allow TF binding and transcriptional initiation [46]. In rice, disruption of the SWI/SNF complex component OsNMCP1 promotes chromatin accessibility and transcription of specific drought-responsive genes, suggesting that the SWI/SNF complex normally maintains a more compact chromatin state at these loci and that its modulation by stress-activated TFs may be a mechanism for rapidly derepressing drought response genes [46]. The CHD3 family chromatin remodeller OsCHR4 represses wax biosynthesis genes and modulates H3K4me3 and H3K27me3 levels at their loci in rice, and loss of OsCHR4 function enhances cuticular wax deposition and drought tolerance, demonstrating that chromatin remodeller activity determines the epigenetic state at stress-adaptive trait loci [46]. In wheat-related Tritipyrum, ATAC-seq combined with RNA-seq identified WRKY26 and HAT5 as TFs whose binding motifs are enriched in open chromatin regions that become more accessible under salt stress, directly connecting WRKY TF binding potential with chromatin accessibility dynamics in a cereal genome under abiotic stress [57]. ATAC-seq analysis in barley revealed that heat stress induces temperature-specific changes in open chromatin regions in crown tissues, with altered chromatin accessibility at the promoters of gibberellin signalling genes associated with corresponding changes in gene expression, establishing that chromatin accessibility is a dynamic and stress-responsive feature of the barley epigenome [58]. Together, these findings suggest that stress-activated TFs direct both histone modification and nucleosome repositioning at target loci, a dual mechanism that likely underlies rapid and robust transcriptional stress responses in cereals [45].

### 5.4. Epigenetic Modulation of TF Binding Site Accessibility

The relationship between TFs and chromatin is not unidirectional. Epigenetic marks established at TF binding sites before or during stress exposure determine whether those sites are accessible to incoming TF proteins, and this accessibility gating is a fundamental mechanism through which epigenetic regulation shapes the selectivity and amplitude of TF-driven transcriptional responses [46]. DNA methylation at gene promoters can physically occlude TF binding by sterically hindering the interaction between TF DNA-binding domains and their cognate cis-regulatory elements, so stress-induced changes in promoter methylation can alter TF occupancy at stress-responsive gene loci without changing TF protein levels [46]. The repressive histone mark H3K27me3, associated with a compact, inaccessible chromatin state, limits TF access to the promoters of genes held in a Polycomb-silenced state, and removal of this mark by Jumonji C domain-containing histone demethylases under stress is required to allow TF-mediated activation of these loci [50]. In rice, the H3K4 methylation reader-chaperone regulator complex activates *OsHKT1;5* expression during salinity stress by maintaining an open chromatin state at its promoter, facilitating TF access to this sodium transporter gene under ionic stress [46]. The histone variant H2A.Z, when present in gene bodies, is associated with a repressed chromatin state in rice, and its eviction under stress is linked to transcriptional derepression, suggesting histone variant dynamics may be another layer of chromatin-based gating of TF activity in cereals [46]. Integration of chromatin accessibility profiling technologies including ATAC-seq with TF binding data and histone modification maps has begun to reveal the genome-wide architecture of TF-epigenome interactions under individual abiotic stresses in rice, maize, wheat, and barley, providing a framework for identifying the chromatin-level regulatory logic of stress responses in these species [57]. Equivalent genome-wide chromatin accessibility profiling under combined stress has not yet been reported in any major cereal, a critical gap in understanding how the TF-epigenome interface reconfigures under simultaneous stresses [45].

### 5.5. Feedback Loops Between Epigenetic Marks and TF Gene Expression

**Figure 4:**
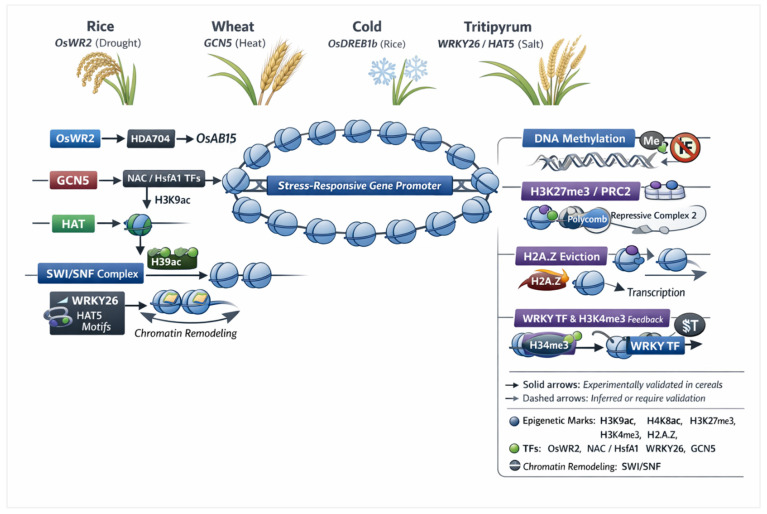
Bidirectional mechanistic interconnections between transcriptional and epigenetic regulatory layers at stress-responsive gene promoters in cereals. The figure depicts the bidirectional coupling between transcription factor networks and epigenetic regulatory machinery at stress-responsive gene promoters under combined abiotic stress conditions in cereals. In the TF-to-epigenome direction, stress-activated TFs recruit histone-modifying enzymes to target gene promoters to alter local chromatin accessibility. In rice, the drought-responsive transcription factor OsWR2 recruits the histone deacetylase HDA704 to the OsABI5 promoter, catalysing deacetylation of H4K8 and modulating ABA-responsive transcription amplitude [51]. In wheat, the histone acetyltransferase GCN5 interacts with NAC-like and HsfA1 transcription factors to regulate thermotolerance gene expression through H3K9 acetylation at target promoters [56]. Cold stress induces hyperacetylation of H3K9 at the OsDREB1b promoter in rice through HAT-mediated chromatin remodelling, demonstrating TF-directed chromatin opening at a cold-responsive locus [56]. TFs also recruit ATP-dependent chromatin remodelling complexes including SWI/SNF to reposition nucleosomes at stress-responsive gene promoters, with WRKY26 and HAT5 binding motifs enriched in salt-stress-induced open chromatin regions in Tritipyrum [57]. In the epigenome-to-TF direction, DNA methylation at gene promoters sterically occludes TF binding domain interactions with cis-regulatory elements, H3K27me3 deposited by Polycomb Repressive Complex 2 restricts TF access to silenced loci until removed by stress-activated demethylases, and histone variant H2A.Z eviction under stress is linked to transcriptional derepression in rice [46]. H3K4me3 levels at WRKY TF gene promoters in rice are dynamically regulated under drought and salinity stress, establishing a feedback loop in which TF activity alters the chromatin state at TF gene loci themselves [50]. Heat stress in barley induces temperature-specific changes in chromatin accessibility at gibberellin signalling gene promoters, demonstrating that chromatin accessibility is a dynamic stress-responsive feature of the cereal epigenome [58]. Solid bidirectional arrows indicate experimentally validated interactions in cereals. Dashed arrows indicate interactions inferred from model organisms or requiring combined-stress-specific validation in cereals.

A further dimension of TF-epigenetic interconnection in cereals is the regulation of TF gene expression itself by epigenetic mechanisms, creating feedback loops in which the activity of a TF determines the epigenetic state of stress-responsive gene loci, which in turn influences TF gene expression through changes in chromatin accessibility at TF promoters [47]. In rice, the bZIP transcription factor OsbZIP23 is regulated in part through ABA-dependent SnRK2-mediated phosphorylation, and its activity alters histone acetylation patterns at its target gene promoters, which may in turn affect the chromatin state at the *OsbZIP23* promoter itself through indirect mechanisms involving shared chromatin-modifying complexes [32]. The methylation state of H3K4 at the promoters of WRKY TF genes in rice is dynamically regulated under drought and salinity stress, and changes in H3K4me3 levels at these loci are associated with altered WRKY expression, suggesting histone methylation establishes a feedback mechanism that modulates TF gene expression under stress [50]. These feedback loops add complexity to the combined-stress response, since a TF activated by one stress signal may alter chromatin state at loci relevant to a second, concurrent stress, creating epigenetically mediated cross-stress interactions that single-stress experiments cannot predict [47]. The molecular identity of these feedback circuits and their genome-wide scope under combined stress conditions in cereals represents an important area for future investigation. The mechanistic framework developed across Section 2, Section 3, Section 4 and Section 5 provides the foundation for Section 6, where CRISPR-based tools for engineering validated targets within this framework are evaluated. Figure 4 illustrates the bidirectional mechanistic connections between TF networks and epigenetic regulatory layers at stress-responsive gene promoters in cereals.

## 6. CRISPR-Based Engineering of Combined Stress Tolerance in Cereals

### 6.1. CRISPR/Cas9 and Emerging Editing Tools in Cereals

**Figure 5:**
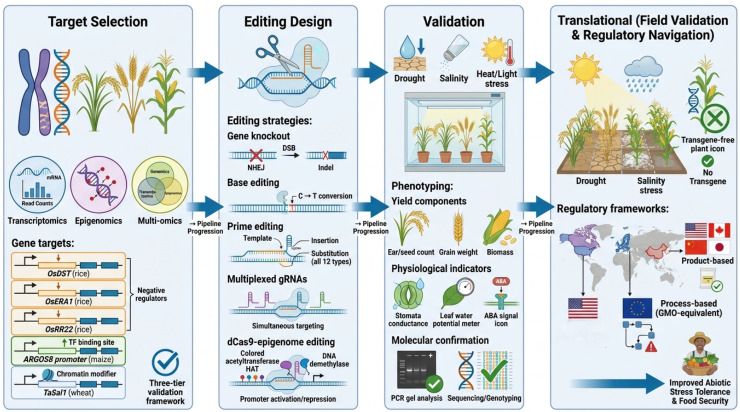
CRISPR-based engineering pipeline for combined abiotic stress tolerance in cereal crops. The figure illustrates the four-stage pipeline from molecular target selection through to field-level validation for CRISPR-based engineering of combined stress tolerance in cereals. In the target selection stage, stress-responsive genes are identified through transcriptomic, epigenomic, and multi-omics analyses under combined stress conditions, with priority given to negative regulators of stress responses whose loss of function releases tolerance mechanisms, and to TF and chromatin modifier genes validated within the three-tier framework [59]. Validated targets include OsDST in rice, whose knockout through CRISPR/Cas9 produces plants with enhanced stomatal closure and tolerance to both drought and salinity [17], OsERA1 in rice, whose loss of function confers enhanced ABA sensitivity and reduced stomatal conductance under drought [17], OsRR22 in rice, whose mutation improves salinity tolerance without agronomic penalties [59], the ARGOS8 promoter in maize, whose replacement with the GOS2 constitutive promoter produced field-validated yield improvements under drought [17], and TaSal1 loci in wheat, whose multiplexed knockout improved drought tolerance under osmotic stress [60]. In the editing design stage, CRISPR/Cas9 is applied for gene knockout through non-homologous end joining, base editing for precise single-nucleotide changes without double-strand breaks, prime editing for insertions and all twelve substitution types, and multiplexed guide RNA strategies for simultaneous editing of multiple stress tolerance loci [59]. Emerging dCas9-based epigenome editing fusing inactive Cas9 to histone acetyltransferase or DNA demethylase effector domains enables targeted chromatin remodelling at stress-responsive loci without sequence alteration [61]. In the validation stage, edited lines are evaluated under single and combined stress conditions in controlled environments, with phenotypic assessment including yield components, physiological stress indicators, and molecular confirmation of edited loci. In the translational stage, transgene-free edited lines are advanced to multi-environment field trials under combined stress conditions, with regulatory pathway navigation varying by jurisdiction, from product-based frameworks in the United States, Canada, China, and Japan to process-based GMO-equivalent regulation in the European Union [59].

CRISPR/Cas9-based genome editing operates through the RNA-guided endonuclease activity of the Cas9 protein, which is directed to a specific genomic locus by a single-guide RNA and introduces a double-strand break that is repaired by either non-homologous end joining, producing insertions or deletions, or homology-directed repair, enabling precise sequence replacement [17]. In cereals, CRISPR/Cas9 has been applied across rice, wheat, maize, barley, and sorghum to modify genes and regulatory elements controlling yield, stress tolerance, and grain quality, and it has become the preferred tool for functional validation of stress-responsive genes identified through the transcriptomic and epigenomic approaches described in Section 3 and Section 4 [59]. Beyond CRISPR/Cas9, base editing technologies fuse a catalytically impaired Cas9 nickase with a cytidine or adenosine deaminase enzyme to convert specific nucleotide bases without generating double-strand breaks, enabling precise single-nucleotide changes at target loci with reduced risk of indel formation [59]. Prime editing extends this precision further by using a reverse transcriptase fused to Cas9 nickase and a prime editing guide RNA that encodes both the target sequence and the desired edit, enabling insertions, deletions, and all twelve types of single-base substitution without requiring double-strand breaks or donor DNA templates [59]. These three editing modalities together provide a toolkit of increasing precision and versatility for engineering stress tolerance targets identified within the TF-epigenetic framework established in this review, and their respective strengths and limitations for combined-stress engineering in cereals are discussed across the following subsections. Figure 5 provides a schematic of the CRISPR-based engineering pipeline from target selection through to field evaluation in cereals. Table 3 summarises the validated CRISPR editing targets, editing tools, stress types addressed, phenotypic outcomes, and experimental validation levels for major cereal species discussed in this section.

### 6.2. Validated CRISPR Targets for Single Stress Tolerance in Cereals

The majority of CRISPR interventions for abiotic stress tolerance in cereals have targeted negative regulators of stress responses, whose loss of function releases suppression of tolerance mechanisms and thereby improves stress performance [59]. In rice, CRISPR/Cas9-mediated knockout of OsDST, which encodes a zinc finger transcription factor that suppresses hydrogen peroxide production in guard cells and promotes stomatal opening, produced plants with increased stomatal closure, improved water retention, and enhanced tolerance to both drought and salinity in the indica mega cultivar MTU1010 [17]. Loss-of-function mutation of OsERA1, which encodes a beta subunit of protein farnesyltransferase that acts as a suppressor of ABA signalling, conferred enhanced ABA sensitivity, reduced stomatal conductance, and improved drought tolerance in rice through CRISPR/Cas9-mediated mutagenesis targeting multiple exons of the gene [17]. In rice, CRISPR/Cas9-induced mutation of OsRR22, encoding a B-type cytokinin response regulator, enhanced salinity tolerance without detectable changes in other agronomic traits under non-stress conditions, demonstrating that targeted negative regulator knockout can improve stress performance with acceptable agronomic trade-offs in a major cereal [59]. In maize, replacement of the endogenous ARGOS8 promoter with the constitutive GOS2 promoter using CRISPR/Cas9 increased ARGOS8 expression, which encodes a negative regulator of ethylene response, and this promoter swap produced field-validated yield improvements under drought stress conditions without yield penalties under well-watered conditions, representing one of the few CRISPR-edited stress tolerance traits validated under field conditions in a cereal crop [17]. In wheat, CRISPR/Cas9 editing of TaDREB2 and TaERF3 improved drought resistance, and multiplexed knockout of multiple TaSal1 loci, which encode inositol polyphosphate 1-phosphatase and act as suppressors of drought stress signalling, improved drought tolerance in edited hexaploid wheat seedlings under polyethylene glycol-imposed osmotic stress conditions [60]. A recurring technical constraint in wheat and other polyploid cereals is homeolog redundancy. Because hexaploid wheat carries three genome copies of most genes, single-genome edits are frequently masked by functionally redundant homeologs on the remaining subgenomes, and complete knockout typically requires simultaneous editing of all A, B, and D copies, which increases both the multiplexing burden and the risk of off-target modification of closely related paralogous sequences elsewhere in the genome [59]. This redundancy issue, rather than editing efficiency per se, is often the limiting factor in translating single-gene CRISPR results from diploid model systems or rice into hexaploid wheat.

### 6.3. Multiplexed CRISPR Editing for Combined Stress Tolerance

Single-gene CRISPR interventions address individual tolerance mechanisms and are inherently limited in their capacity to improve tolerance to combined stresses that require simultaneous engagement of multiple regulatory pathways [59]. Multiplexed CRISPR editing, in which multiple guide RNAs targeting different genes are expressed simultaneously from a single construct, enables the coordinated modification of several stress tolerance loci in a single transformation event and represents a more appropriate strategy for engineering combined stress tolerance [59]. In rice, simultaneous CRISPR/Cas9 knockout of four negative regulator genes, *OsMads26*, *OsBsr-d1*, *OsELF3-2*, and *OsERF922*, produced quadruple mutant lines that exhibited substantially higher tolerance to both drought and salt stress than wild-type plants, while also displaying increased grain yield of 17.35% to 21.95%, demonstrating that coordinated multi-gene editing can simultaneously improve stress tolerance and yield in rice [65]. In maize, co-expression of ZmVPP1, encoding a vacuolar H^+^-pyrophosphatase, with ZmNAC111, encoding a drought-responsive NAC transcription factor, through CRISPR-assisted regulatory engineering conferred robust drought resistance by enhancing the plant’s integrated stress response through both vacuolar proton pumping and transcriptional activation of stress-responsive genes [17]. A well-documented risk with constitutively expressed stress-tolerance edits, illustrated by transgenic rather than CRISPR studies to date, is growth retardation. Constitutive overexpression of DREB1A-type factors consistently improves stress tolerance but also represses growth-promoting pathways under non-stress conditions, producing a yield penalty that can offset the tolerance gain in favourable seasons [10]. In *Arabidopsis*, co-expressing DREB1A with the growth-promoting genes *GA5* or *PIF4*, whose own expression is normally repressed under drought, substantially reduced this growth penalty while preserving stress tolerance, indicating that pairing a stress-tolerance edit with a growth-derepression edit is a mitigation strategy that could in principle be adapted to multiplexed CRISPR designs in cereals, though this specific approach has not yet been implemented via genome editing in any cereal species [67]. These multiplexed approaches align with the three-tier framework established in this review, as they simultaneously target TF-level regulatory genes identified in Section 3, thereby amplifying the transcriptional stress response through precise genetic modification of multiple nodes in the TF network [12]. The development of multiplexed CRISPR strategies specifically designed to address the combined stress signalling crosstalk described in Section 2 remains at an early stage, and the field currently lacks validated examples in which guide RNA targets have been selected based on their specific roles in combined rather than individual stress responses in cereals [16].

### 6.4. CRISPR-Based Epigenome Editing for Stress Tolerance

An emerging dimension of CRISPR-based engineering that directly engages the epigenetic regulatory tier described in Section 4 and Section 5 is epigenome editing, in which a catalytically inactive dCas9 protein is fused to epigenetic effector domains to deposit or remove specific histone marks or DNA methylation at targeted genomic loci without altering the underlying DNA sequence [61]. By fusing dCas9 with histone acetyltransferase catalytic domains, it is possible to locally increase H3K9ac or H3K27ac levels at stress-responsive gene promoters, converting repressive chromatin into an accessible state that facilitates TF binding and transcriptional activation [61]. In *Arabidopsis thaliana*, a dCas9 fused to a histone acetyltransferase catalytic domain was used to acetylate histones at the AREB1 promoter, enhancing AREB1 expression, promoting stomatal closure under drought, and improving plant survival under water deficit conditions, suggesting that targeted epigenetic activation of ABA-responsive TF genes may be a viable strategy for improving drought tolerance without introducing sequence changes [61]. This approach remains unvalidated in any major cereal species. Translating it from *Arabidopsis* to polyploid wheat compounds the epigenome-editing challenges (delivery efficiency, off-target epigenomic effects, mark stability across cell divisions and generations) with the same homeolog-redundancy problem described in Section 6.2, since epigenetically silencing one subgenome copy of a gene may be functionally masked by unedited copies on the other two subgenomes [61]. The dCas9-SunTag system, which amplifies epigenetic effector recruitment through a repetitive peptide scaffold, offers improved efficiency of epigenetic mark deposition at target loci and may help overcome some of the sensitivity limitations of single effector domain fusions in cereals [61]. The combination of epigenome editing with the mechanistic knowledge of TF-chromatin modifier interactions described in Section 5 provides a conceptual framework for designing targeted interventions that simultaneously modulate TF activity and chromatin accessibility at combined-stress-responsive loci, though direct experimental implementation of this strategy in field-relevant cereal systems has not yet been reported [45].

### 6.5. Field Performance and Regulatory Considerations

The translation of CRISPR-edited stress tolerance traits from controlled laboratory conditions to field performance in cereals presents challenges that are distinct from those of molecular design and are frequently underestimated in the research literature [16]. Stress tolerance phenotypes validated under controlled greenhouse or growth chamber conditions, where a single stress is applied at a defined intensity and developmental stage, may not translate directly to field conditions where multiple stresses co-occur at variable intensities and interact with soil microbiome composition, agronomic management practices, and genotype-specific background effects [10]. The ZmARGOS8 promoter replacement in maize represents a notable exception in that drought tolerance improvement was validated under field conditions across multiple environments, but this remains one of only a small number of CRISPR-edited stress tolerance traits with multi-environment field data in any cereal [17]. A comparable multi-environment field result exists for conventional transgenic overexpression rather than CRISPR editing. Constitutive expression of the soybean transcription factor GmDREB1 in two wheat cultivars produced grain yield increases of 4.79% to 18.43% under limited-irrigation and non-irrigated conditions across three growing seasons and three production sites in the North China Plain, without a significant yield penalty under well-watered conditions [68]. This example illustrates both the risk-benefit trade-off relevant to CRISPR translation and the scale of field validation that CRISPR-edited stress-tolerance traits in cereals have yet to match. The wheat result was achieved by conventional transgenesis, not genome editing, and no CRISPR-edited combined-stress trait in any cereal has been tested at a comparable multi-year, multi-site scale. The first is the possibility of a growth or yield penalty under non-stress conditions, as discussed in Section 6.3. The second is the possibility that homeolog redundancy or off-target effects in polyploid wheat mask or complicate the intended phenotype, as discussed in Section 6.2. The third is the possibility that a single-gene or single-pathway edit validated under one stress fails to confer benefit, or confers a cost, when the crop encounters a different or combined stress in the field. Editing strategies that target genes with well-characterised trade-off profiles, such as OsRR22 and the maize ARGOS8 promoter swap, where prior evidence indicates minimal growth penalty, are lower risk than constitutively active DREB2-type edits, where a growth penalty is well documented, though direct evidence for this trade-off comes mainly from transgenic overexpression rather than CRISPR-specific studies [10]. The global regulatory landscape for CRISPR-edited crops differs substantially across jurisdictions, with some countries including the United States, Japan, Canada, China, and several South American nations adopting product-based regulatory frameworks that exempt transgene-free genome-edited plants from the most stringent GMO oversight requirements, while the European Union continues to apply process-based regulations that have historically classified directed mutagenesis products as GMOs [59]. China issued safety evaluation guidelines for transgene-free genome-edited plants in 2022 and has conducted field trials of CRISPR-edited rice varieties, representing significant progress toward commercialisation of edited cereal crops in Asia [59]. These regulatory divergences carry socio-economic consequences beyond simple market access. The cost and delay associated with process-based regulatory approval disproportionately burden public-sector and smallholder-oriented breeding programmes relative to well-resourced multinational developers, meaning that the jurisdictions where combined-stress tolerance is most agronomically urgent, including much of sub-Saharan Africa and South Asia, do not necessarily have the fastest regulatory pathway to deploy it. These regulatory divergences create barriers to global deployment of CRISPR-edited stress-tolerant cereals and mean that the pathway from laboratory validation to farmer-accessible varieties varies substantially across production regions [59]. The knowledge gaps and translational barriers that most critically constrain the deployment of the three-tier framework developed in this review are addressed in Section 7.

## 7. Challenges, Knowledge Gaps, and Future Directions

### 7.1. The Fundamental Gap in Combined-Stress Molecular Research

The most consequential knowledge gap constraining progress in cereal stress tolerance research is the near-total absence of molecular studies specifically designed to characterise responses to combined, simultaneously applied stresses under field-relevant conditions [1]. As established in Section 2, the transcriptional, hormonal, and epigenetic responses activated by combined stresses in cereals are qualitatively distinct from those elicited by individual stresses and cannot be predicted by summing or overlaying single-stress datasets [10]. Yet the vast majority of studies characterising TF functions, epigenetic dynamics, and CRISPR target validation in cereals reviewed across Section 3, Section 4, Section 5 and Section 6 were conducted under single-stress conditions in controlled environments, meaning that the mechanistic framework presented in this review rests on an evidence base that was largely not generated under conditions representative of field-level combined stress exposure [4]. Closing this gap requires combined-stress treatments applied at agronomically realistic intensities, developmental stages, and timings, with molecular phenotyping resolved enough to capture the qualitatively novel responses that emerge under simultaneous stress [1]. The field’s current reliance on individual stress experiments has produced extensive knowledge about stress-specific pathways but has left the regulatory logic of combined-stress integration, including which TFs, chromatin modifiers, and epigenetic marks are specifically induced by the combination rather than by either stress alone, poorly defined in every major cereal species [22].

### 7.2. Species-Specific Knowledge Gaps

The molecular characterisation of combined stress responses is substantially more advanced in rice and wheat than in maize, barley, and sorghum, creating an uneven knowledge base that limits the generalisability of the three-tier framework proposed in this review [4]. In rice and wheat, genome sequences, transcriptome atlases, TF binding datasets, and epigenome maps are well-developed and have enabled the mechanistic studies described in Section 3, Section 4 and Section 5. In barley, combined stress transcriptomics has identified large-scale TF gene expression changes under drought and heat, as described in Section 3, but the functional characterisation of individual barley TFs and chromatin modifiers under combined stress conditions lags considerably behind rice and wheat [11]. In sorghum, which possesses inherent tolerance to drought, heat, and salinity that makes it a potentially valuable model for combined stress research, the molecular mechanisms underlying its superior stress tolerance have been examined primarily through transcriptomic, proteomic, and metabolomic analyses under individual stresses, and the TF-epigenetic regulatory architecture of its combined stress response has not been systematically characterised [4]. The development of sorghum as a comparative model for combined stress tolerance, using its natural resilience as a reference point for identifying regulatory circuits that are absent or less active in stress-sensitive cereals, represents a high-priority opportunity that the field has not yet fully exploited [4]. In maize, the large and complex genome creates specific technical challenges for chromatin accessibility profiling and epigenome mapping under combined stress conditions, and the development of single-cell multi-omics approaches to resolve cell-type-specific TF-epigenome interactions under combined stress conditions in maize root and leaf tissues is an important emerging priority [13].

### 7.3. Absence of Combined Stress Epigenomic Data in Cereals

As noted in Section 4, genome-wide epigenomic profiling under combined stress conditions in any major cereal species has not been reported as of the time of writing this review [45]. The existing epigenomic datasets for cereals under abiotic stress have been generated under single-stress conditions, and the combined-stress epigenome, including the DNA methylation landscape, histone modification patterns, chromatin accessibility profile, and non-coding RNA expression networks that emerge when two or more stresses are applied simultaneously, remains entirely uncharacterised at the whole-genome level in rice, wheat, maize, barley, or sorghum [45]. Generating such datasets using bisulphite sequencing for DNA methylation, ChIP-seq or CUT-and-Tag for histone modifications, ATAC-seq for chromatin accessibility, and small RNA-seq for non-coding RNA populations, all applied simultaneously under combined drought-heat, salinity-heat, and drought-salinity treatments in field-relevant cereal genotypes, represents a scientifically urgent and tractable experimental priority [69]. The integration of these epigenomic datasets with TF binding and transcriptome data from combined stress experiments would enable, for the first time, a genome-wide view of how the TF-epigenome interconnections described in Section 5 are specifically reconfigured under combined stress conditions in cereals [45].

### 7.4. Multi-Omics Integration and AI-Driven Target Identification

The complexity of combined stress responses in cereals, which involves the simultaneous modulation of thousands of genes across multiple regulatory tiers, necessitates analytical frameworks that go beyond the interpretation of individual datasets [69]. Multi-omics integration, combining genomic, transcriptomic, proteomic, metabolomic, and epigenomic data from the same biological material under defined combined stress treatments, is emerging as the most powerful strategy for identifying the causal regulatory nodes that determine combined stress tolerance outcomes [13]. In wheat, a meta-transcriptomic analysis integrating 100 RNA-seq datasets under heat, cold, drought, and salt stress identified BES1/BZR1 brassinosteroid-responsive TFs and glycosyl hydrolases as hub genes integrating multi-stress responses, demonstrating the power of large-scale data integration for prioritising targets that would not emerge from individual single-stress experiments [44]. Artificial intelligence and machine learning approaches, applied to multi-omics data from diverse cereal genotypes under combined stress conditions, offer the potential to identify non-obvious regulatory interactions, predict the phenotypic consequences of specific TF or chromatin modifier perturbations, and prioritise CRISPR editing targets with the highest probability of improving combined stress tolerance without yield penalties [70]. AI-powered integrative frameworks incorporating epigenomic, transcriptomic, and field phenotypic data remain at an early stage in cereal stress biology, and their potential to accelerate deployment of the three-tier framework proposed here is not yet realised [70].

### 7.5. Translational Barriers and Future Breeding Priorities

The translation of mechanistic insights from Section 2, Section 3, Section 4, Section 5 and Section 6 into field-deployable cereal varieties tolerant to combined abiotic stresses faces several interconnected barriers that extend beyond the molecular knowledge gaps addressed above [16]. The polygenic nature of combined stress tolerance means that even well-validated single-gene CRISPR edits, or individual epigenetic interventions, are unlikely to produce the robust and durable tolerance improvements required under field conditions, where stress combinations vary in intensity, timing, and developmental stage interaction [59]. Multiplex CRISPR editing strategies that simultaneously target multiple nodes in the TF-epigenetic network described in this review, informed by multi-omics target identification and validated under combined stress field trials, represent the most promising near-term translational pathway for this framework [65]. The integration of CRISPR-based sequence editing with epigenome editing using dCas9-effector fusions to simultaneously modify TF network components and their chromatin environments represents a longer-term but potentially more durable approach, as it addresses both the genetic and epigenetic dimensions of combined stress tolerance in a single engineering event [61]. Epiallele-based breeding, in which natural or induced epigenetic variants at stress-responsive loci are identified, characterised, and introgressed into elite cereal backgrounds through marker-assisted selection incorporating epigenetic markers alongside SNP markers, provides a complementary and non-transgenic route to deploying the epigenetic regulatory insights from Section 4 in breeding programmes [45]. Figure 2 summarises the TF regulatory networks across cereal species discussed in Section 3 and Section 7, Figure 3 illustrates the epigenetic mechanisms and stress memory circuits addressed in Section 4 and Section 7, Figure 4 depicts the TF-epigenome interconnections that are priorities for combined-stress characterisation, and Figure 5 maps the CRISPR engineering pipeline from target selection through field validation that remains to be fully implemented for combined stress targets in cereals. Table 3 identifies the validated CRISPR targets discussed in Section 6 and their potential extension to combined stress scenarios as a future research priority (Table 3). The resolution of the knowledge gaps identified in this section is a prerequisite for deploying the three-tier integrated framework in crop improvement practice, and the specific scientific priorities are reinforced in the Conclusion.

## 8. Conclusions

Cereal crops face an accelerating threat from combined abiotic stresses that co-occur under field conditions with increasing frequency and severity. Yield losses from simultaneous drought, heat, salinity, and cold are consistently more severe than those caused by any individual stress, and the molecular responses they elicit are qualitatively distinct from single-stress responses in ways that cannot be predicted from existing single-stress datasets. This review has developed a three-tier integrated framework for understanding and engineering combined abiotic stress tolerance in major cereal crops, and to our knowledge this is the first synthesis to explicitly integrate transcription factor network dynamics, epigenetic priming, and CRISPR-based genome and epigenome editing into a single mechanistic account of this problem, rather than treating these regulatory layers as separate studies. The first tier encompasses the transcription factor networks together with the osmoprotectant and antioxidant defence systems they regulate, through which combined stress signals are translated into coordinated transcriptional reprogramming. TF families including bZIP, WRKY, NAC, AP2/ERF, DREB, MYB, and HSF respond simultaneously under combined stress. Their network-level interactions, rather than individual activations, determine the tolerance phenotype, though this regulatory logic remains incompletely characterised in barley and sorghum. The second tier addresses the epigenetic regulatory layer that gates TF binding site accessibility, encodes stress memory, and may transmit stress-adaptive states across generations, though genome-wide epigenomic profiling under combined stress has not yet been conducted in any major cereal. The third tier examines CRISPR-based tools that engineer validated targets within the first two tiers. CRISPR/Cas9, base editing, prime editing, and dCas9-based epigenome editing provide a precision toolkit for cereals, but homeolog redundancy in polyploid wheat, growth penalties under constitutive expression, and the persistent gap between greenhouse and multi-environment field validation constrain near-term deployment, and multiplexed strategies targeting combined-stress-specific nodes remain at an early stage. The three tiers are mechanistically coupled rather than independent, with TF activity shaping the epigenome, epigenetic states gating TF access, and both layers providing validated engineering targets. Progress will require combined-stress experimental designs, multi-omics data integration, and field-validated translation across all three tiers simultaneously. This framework provides the conceptual and mechanistic foundation for that research programme.

## Figures and Tables

**Table 1:** Key transcription factor families involved in abiotic stress responses in major cereal crops. Evidence levels are defined as follows. L1 indicates direct functional validation in the target cereal species under stress conditions. L2 indicates validation in a heterologous cereal system. L3 indicates validation in *Arabidopsis thaliana* using a cereal-derived gene.

TF Family	DNA-Binding Domain	Gene	Stress Type	Regulation	Cereal Species	Evidence Level	Reference
bZIP/AREB	bZIP domain binding ACGT ABRE core motif	*OsbZIP23*	Drought, salinity	Positive	*O. sativa*	L1	[30]
bZIP/AREB	bZIP domain binding ACGT ABRE core motif	*OsbZIP71*	Drought, salinity	Positive	*O. sativa*	L1	[31]
bZIP/AREB	bZIP domain binding ACGT ABRE core motif	*TaAREB3*	Drought	Positive	*T. aestivum*	L3	[32]
bZIP/AREB	bZIP domain binding ACGT ABRE core motif	*TabZIP8-7A*	Drought	Positive	*T. aestivum*	L1	[32]
bZIP/AREB	bZIP domain binding ACGT ABRE core motif	*ZmbZIP68*	Cold	Negative	*Z. mays*	L1	[32]
WRKY	WRKYGQK heptapeptide binding W-box TTGACC/T	*ZmWRKY40*	Drought, salinity, heat	Positive	*Z. mays*	L1	[33]
WRKY	WRKYGQK heptapeptide binding W-box TTGACC/T	*ZmWRKY82*	Drought	Positive	*Z. mays*	L1	[34]
WRKY	WRKYGQK heptapeptide binding W-box TTGACC/T	*OsWRKY5*	Drought	Negative	*O. sativa*	L1	[35]
WRKY	WRKYGQK heptapeptide binding W-box TTGACC/T	*TaWRKY1-2D*	Drought	Positive	*T. aestivum*	L2	[36]
NAC	NAC domain binding NAC recognition sequence	*OsNAC092*	Drought	Negative	*O. sativa*	L1	[37]
NAC	NAC domain binding NAC recognition sequence	*OsNAC25*	Drought, heat, salinity, cold	Positive	*O. sativa*	L1	[38]
NAC	NAC domain binding NAC recognition sequence	*TaNAC071-A*	Drought	Positive	*T. aestivum*	L1	[39]
AP2/ERF	AP2 domain binding GCC box element	*TaEREBP1-L*	Drought and heat combined	Positive	*T. aestivum*	L1	[40]
DREB	AP2 domain binding DRE/CRT element TACCGACAT	*TaDREB2*	Drought, salinity	Positive	*T. aestivum*	L1	[11]
DREB	AP2 domain binding DRE/CRT element TACCGACAT	*DREB2A/2C*	Drought and heat combined	Positive	*T. aestivum*	L1	[40]
HSF	HTH domain binding HSE nGAAn consensus	*ZmHsf05*	Drought, heat	Positive	*Z. mays*	L2	[41]
HSF	HTH domain binding HSE nGAAn consensus	*TaHSFC3B*	Drought	Positive	*T. aestivum*	L2	[42]
MYB	MYB repeat domain binding MYB recognition sequence	Multiple members	Drought, salinity, heat, cold combined	Variable	*Z. mays*	L1	[33]

**Table 2:** Epigenetic regulators and stress memory mechanisms characterised in cereal crops under abiotic stress. Evidence levels follow the same scale as Table 1.

Epigenetic Mechanism	Enzyme or Complex	Target Locus or Category	Stress Condition	Cereal Species	Evidence Level	Reference
CG DNA methylation	MET1 methyltransferase	Stress-responsive gene promoters’ genome-wide	Drought and salinity, single stress only	*O. sativa, T. aestivum*	L1	[48]
CHH DNA methylation	DRM2 via RdDM pathway	Transposable elements near stress genes	Heat, single stress only	*O. sativa, Z. mays*	L1	[48]
Promoter DNA methylation	MET1 and DRM2	NAC111 promoter	Drought, single stress only	*T. aestivum*	L1	[22]
Genome-wide methylation dynamics	DRM2 and MET1	Genome-wide differentially methylated regions	Drought, single stress only	*Z. mays*	L1	[48]
H3K4me3 deposition	SDG methyltransferases	HKT1;5 promoter and coding region	Saline-alkaline, single stress only	*O. sativa*	L1	[49]
H3K9ac deposition	Histone acetyltransferases	Hsf-01 and Hsf-15 promoters	Heat, single stress only	*Z. mays*	L1	[45]
H3K9me2 reduction	Histone demethylases	Heat-responsive gene loci	Heat, single stress only	*O. sativa*	L1	[45]
H3K27me3 removal	JmjC domain demethylases	Stress-responsive Polycomb-silenced loci	Stress-induced derepression	*O. sativa, T. aestivum*	L1	[50]
Bivalent H3K4me3 and H3K27me3	PRC2 and SDG complexes	Poised stress-responsive gene promoters	Multiple stresses, single stress only	*O. sativa*	L1	[50]
H4K8 deacetylation	HDA704 recruited by OsWR2	OsABI5 promoter	Drought, single stress only	*O. sativa*	L1	[51]
miRNA regulation	DICER-LIKE proteins	Stress-responsive mRNA targets	Drought, heat, salinity, cold	*O. sativa, T. aestivum, Z. mays*	L1	[52]
siRNA-directed RdDM	RDR2, DICER-LIKE 3, DRM2	Transposable elements and euchromatin boundaries	Drought, single stress only	*Z. mays, O. sativa*	L1	[53]
lncRNA-mediated chromatin regulation	Polycomb complex recruitment	TCONS_00028567 target loci	Drought memory, single stress only	*O. sativa*	L1	[48]
Somatic stress memory	H3K4me3 and H3K9ac persistence	Memory gene promoters	Drought priming	*O. sativa, T. aestivum, Z. mays*	L1	[47]
Transgenerational epigenetic inheritance	MET1, DRM2, H3K27me3	ABA-related and auxin-related gene loci	Drought, salt, heat	*T. aestivum, O. sativa*	L1	[47]
Reproductive epigenetic reprogramming	RdDM and DNA methyltransferases	Male gametophyte development loci	Multiple abiotic stresses	*O. sativa, Z. mays, T. aestivum*	L1	[54]

**Table 3:** CRISPR-based genome editing targets for abiotic stress tolerance improvement in cereal crops. Editing tool abbreviations are defined as follows. Cas9 indicates CRISPR/Cas9-mediated indel generation via non-homologous end joining. BE indicates base editing. PE indicates prime editing. Cas9-PR indicates CRISPR/Cas9-mediated promoter replacement. Multi Cas9 indicates multiplexed CRISPR editing targeting two or more loci simultaneously. dCas9-HAT indicates catalytically inactive dCas9 fused to a histone acetyltransferase effector domain. Validation levels are defined as follows. V1 indicates validation under controlled laboratory or greenhouse conditions. V2 indicates validation under field conditions in at least one environment. V3 indicates validation under combined stress conditions.

Target Gene	Molecular Function	Editing Tool	Stress Type	Phenotypic Outcome	Cereal Species	Validation Level	Reference
*OsDST*	Zinc finger TF suppressing guard cell hydrogen peroxide production	Cas9	Drought and salinity	Enhanced stomatal closure, improved water retention, reduced Na accumulation	*O. sativa*	V1	[62]
*OsERA1*	Beta subunit of protein farnesyltransferase suppressing ABA signalling	Cas9	Drought	Enhanced ABA sensitivity, reduced stomatal conductance, improved survival	*O. sativa*	V1	[63]
*OsRR22*	B-type cytokinin response regulator	Cas9	Salinity	Improved salinity tolerance without agronomic penalties	*O. sativa*	V1	[64]
*OsbHLH024*	bHLH TF modulating ion transporter expression	Cas9	Salinity	Increased chlorophyll content and shoot biomass under salt stress	*O. sativa*	V1	[59]
*OsMads26, OsBsr-d1, OsELF3-2, OsERF922*	Negative regulators of defence and stress responses	Multi Cas9	Drought and salinity combined	Enhanced tolerance to drought and salt stress, grain yield increase of 17 to 22%	*O. sativa*	V1	[65]
*ARGOS8* promoter	Negative regulator of ethylene response	Cas9-PR	Drought	Improved grain yield under field drought conditions without yield penalty under well-watered conditions	*Z. mays*	V2	[66]
*ZmVPP1* and *ZmNAC111*	Vacuolar H^+^-pyrophosphatase and drought-responsive NAC TF	Multi Cas9	Drought	Enhanced integrated drought resistance through vacuolar proton pumping and TF-mediated gene activation	*Z. mays*	V1	[17]
*TaDREB2* and *TaERF3*	DREB and ERF TFs mediating drought response	Cas9	Drought	Improved drought resistance	*T. aestivum*	V1	[17]
*TaSal1* multiple homeoloci	Inositol polyphosphate 1-phosphatase suppressing drought signalling	Multi Cas9	Drought	Improved seedling survival under osmotic stress	*T. aestivum*	V1	[60]
*TaHAG1*	Histone acetyltransferase modulating chromatin accessibility	Cas9	Salinity	Enhanced salt tolerance through chromatin-level regulation	*T. aestivum*	V1	[59]
*AREB1* promoter region	ABA-responsive bZIP TF controlling stomatal closure	dCas9-HAT	Drought	Enhanced AREB1 expression, improved stomatal closure and survival under water deficit; validated in *Arabidopsis thaliana* only, with cereal validation pending	*Arabidopsis thaliana* model	V1 model organism only	[61]

## Data Availability

No new data were created or analyzed in this study. Data sharing is not applicable to this article.
